# Towards optimising local reviews of severe incidents in maternity care: messages from a comparison of local and external reviews

**DOI:** 10.1136/bmjqs-2015-004960

**Published:** 2016-03-24

**Authors:** Anjali Shah, Bryn Kemp, Susan Sellers, Lisa Hinton, Melanie O'Connor, Peter Brocklehurst, Jenny Kurinczuk, Marian Knight

**Affiliations:** 1 National Perinatal Epidemiology Unit, Nuffield Department of Population Health, University of Oxford, Oxford, UK; 2 Department of Obstetrics and Gynaecology, University Hospitals Bristol NHS Trust, Bristol, UK; 3 Health Experiences Research Group, Department of Primary Health Care Sciences, Oxford University, Oxford, UK; 4 Institute for Women's Health, University College London, London, UK

**Keywords:** Near miss, Audit and feedback, Incident reporting, Obstetrics and gynecology, Womens health

## Abstract

**Background:**

Detailed local case review is commonly used as a strategy to improve care. However, recent reports have highlighted concerns over quality of local reviews in maternity care. The aim of this project was to describe the methods used for conducting local reviews of care of women with severe maternal morbidity, and to compare lessons identified for future care through external and local reviews.

**Methods:**

Thirty-three anonymised clinical records from women with severe maternal morbidities were obtained, together with the report of the local review of their care. The methodology used for the local reviews was described, including specific tools used, team members involved, their disciplines, report format and whether an action plan with recommendations for audit was produced. Multidisciplinary external reviewers considered the records using a standard confidential enquiry approach. A thematic analysis of lessons learned from the two approaches was undertaken.

**Results:**

A formal report of the local review was produced for 11/33 cases; 4 of these used root cause analysis. A further 12 local reviews consisted of a group discussion with output noted in a spreadsheet; 5 consisted of a timeline with good practice points and 5 had no formal review. Patients were involved in five local reviews; only one was multidisciplinary. Action plans were recorded in 14 local reviews; 3 of these included a recommendation to audit the proposed changes. External reviews identified additional messages for care and highlighted aspects of good care in every case, whereas only 55% (n=18) of local reviews identified good care (p<0.0005).

**Conclusions:**

The quality of local reviews can clearly be improved. Very few of the reviews involved patients. Local reviews should be multidisciplinary, generate an action plan, and the implementation of recommendations should be audited. Improvements in local reviews may be achieved by standardised training or development of national protocols.

## Introduction

Methodical and detailed case review is commonly used as a strategy to improve care, including care of pregnant women^1^ through documenting the number and causes of morbidity and mortality, and through identifying preventable factors.^2^ ^3^ Two approaches have been taken nationally in the UK to learning from adverse incidents in maternity care: external anonymised case review (Confidential Enquiries),^4^ defined as an independent systematic multidisciplinary anonymous investigation which identifies the causes and avoidable or remediable factors associated with them,^5^ and local (facility-based) reviews using different tools such as root cause analysis.^5^ Root cause analysis is a structured methodology used to identify the most likely underlying causes of incidents within an organisation. The aim of both is to formulate solutions to prevent the incident occurring again to protect the health and safety of the public and encourage a culture of openness.^6^ However, reports following recent high-profile systematic failures in maternity care have noted that local reviews of serious incidents have not always identified the key messages to improve care.^7^

No national strategy exists in the UK for local learning from near-miss maternal morbidity, and little is known about the nature of approaches being used in different hospitals. The recent UK Confidential Enquiry into Maternal Deaths and Morbidity, which examined the care of women who died from sepsis, as well as women who survived septic shock, found that local reviews had not been undertaken for most cases, and on several occasions it was unclear whether, when undertaken, a local review had been multidisciplinary and what specific method had been used.^4^ The aim of this project was to describe the methods used for conducting local reviews of the care of women with severe morbidity in pregnancy, to compare the lessons identified for future care through external review (confidential enquiry) and local review, and to estimate the additional costs associated with external review, in order to inform development of a strategy for optimising review of serious incidents in maternity care.

## Methods

Six sites were randomly selected following stratification from all National Health Service (NHS) consultant-led maternity units in England (two large teaching hospital units (5000+ deliveries per year), two medium units (2000–4999 deliveries per year) and two small units (up to 1999 deliveries per year)), and on the basis of their Clinical Negligence Scheme for Trusts (CNST) level at the time of selection^8^ (one of each size with CNST level 1, the basic risk management level, and one of each size with CNST level 3, the highest risk management level). Two of the initially selected sites declined to participate; resampling was therefore carried out to identify two further units which both agreed to participate. Sites were located in the North East, Midlands and South East of England.

Each unit provided a list of all serious incidents in maternity care that had triggered the requirement for a local review process during the previous 6 months (listed in table 1). From these, incidents of direct (obstetric) near-miss maternal morbidity were identified (listed as included conditions in table 1). From these lists of near-miss maternal morbidity incidents, six cases were randomly selected from each unit, and full sets of clinical notes, together with their local review reports, were requested. In total, 33 sets of anonymised clinical records were obtained and reviewed; the remaining 3 sets of notes were unavailable. The methodology used for the local reviews was identified from the reports, in terms of any specific tools used, such as root cause analysis, the team members involved and their disciplines. The reports were analysed to determine whether an action plan was present, and whether this included plans to audit the results of any recommended actions.

**Table 1:** Conditions leading to local review processes during the 6 months of the study

Conditions included	Conditions excluded
Severe haemorrhage (>1500 mL)	Shoulder dystocia
Amniotic fluid embolism	Other maternal incidents
Cardiac arrest	Violation of local protocol
Eclampsia/pre-eclampsia	Retained swab or instrument
Uterine rupture	Medication error
Uterine inversion	Organisational incidents
Severe maternal sepsis	Third-/fourth-degree tears
Hysterectomy	Unsuccessful forceps/ventouse
Placental accreta	Readmission of mother
Placental praevia	
Pulmonary embolism	
Major anaesthetic complications	
Intensive care admission (maternal)	
Acute fatty liver	

Reviews undertaken for near-miss maternal morbidity conditions were included in the study; other incidents were excluded.

External reviews were undertaken using a standard format as used by the Confidential Enquiry into Maternal Deaths.^4^ ^9^ As part of this standard approach, assessors were required to refer to national evidence-based guidelines when commenting on care. All 11 external reviewers, comprising 5 obstetricians, 4 midwives and 2 anaesthetists, received an online training session prior to undertaking assessments. Two obstetricians, a midwife and two anaesthetists assessed the care of each woman; five reviewers thus examined each woman's care. Each primary assessor completed an independent review of the woman's care, highlighting the lessons to be learned to improve care in the future. This was checked by a second assessor in the relevant specialty. Expert assessors were located throughout England; to maintain anonymity, assessors were only asked to review the care of women who had been cared for outside their region. The assessment process and all individual findings were strictly confidential; all assessors were required to sign a confidentiality agreement. Upon completion of all case assessments and reports, each assessor was asked to complete a short questionnaire describing their experience of the process and estimating the time it required to assess each case. The administrative time required to liaise with units, photocopy, anonymise and scan each record and liaise with external assessors was also captured. The average administrative staff and assessor time required for each case were then calculated, and the cost estimated using published salary scales (national spinal point 18 for administrative staff, consultant medical salary scale, band 7 midwife scale).

Qualitative and quantitative approaches were used to compare the lessons learned from local and external reviews.^10^ ^11^ Emerging themes among the lessons learned were initially identified through reading and re-reading the reviews, and a coding frame was constructed and agreed upon by four authors. The data were then coded by three authors and checked by a fourth author. NVIVO 10 software was used to facilitate the analysis.^12^ Once the themes had been identified, the number of reviews identifying each theme was quantified and compared using χ^2^ test or Fisher’s exact test as appropriate.

Following completion of the analysis of local and external reviews, results of the study/comparison were presented to the staff at the six participating units, and local staff were asked to complete a questionnaire detailing their perspectives on the process of undertaking local reviews and the feedback from the external reviews.

## Results

The care of 33 women with near-miss maternal morbidities was assessed by the confidential enquiry panel; the majority of women (73%) had severe haemorrhage (>1500 mL blood loss) (table 2).

**Table 2:** Numbers of near-miss maternal morbidity conditions reviewed in the study*

Conditions	Numbers
Severe haemorrhage (>1500 mL)	24
Amniotic fluid embolism	1
Cardiac arrest	1
Eclampsia/pre-eclampsia	3
Uterine rupture	3
Uterine inversion	2
Severe maternal sepsis	4
Hysterectomy	6
Placental accreta	1
Placental praevia	2
Pulmonary embolism	0
Major anaesthetic complications	1
Intensive care admission (maternal)	3
Acute fatty liver	1

*Several cases involved more than one type of near-miss maternal morbidity condition; thus, the total number of conditions exceeds 33, even though the actual number of cases was 33.

### Local review processes

All the cases had triggered the requirement for a local review, that is, local guidance had indicated that a review should be carried out. However, in five cases, the records had either been reviewed by the individual responsible for conducting local reviews, who had felt that there were no lessons to be learned and a formal review was not required, or the staff responsible for conducting a local review had not been able to because they did not have time capacity to review all the cases which triggered a review requirement. Thus local reviews were carried out for only 28 of 33 women (85%). Methods and reports of the outcome of local reviews were very varied. A formal report of the local review process and outcome had been written in 11 cases; 4 of these local reviews used root cause analysis. For 12 cases, a local review group had discussed the incident, and a summary of the outcome had been noted in a spreadsheet. For an additional five cases the documented local review constituted a timeline of events with brief notes on evidence of good care and if issues had occurred.

The categories of staff involved in conducting reviews varied between units. An individual midwife conducted four reviews, a group of midwives conducted two reviews, and five reviews involved both obstetricians and midwives. One of these five reviews included an anaesthetist, and one included midwifery trainees. It was not possible to identify the specialty or grade of reviewers in 17 local review reports nor was it possible to identify how many reviewers participated. There was patient involvement in five local reviews. In these cases it was documented that the woman concerned had asked the review group to consider specific questions about her care, and that she was later notified of the group's conclusions and action plans.

In the local review reports, action plans had been written for 14 cases. The conclusions from three local review reports included a recommendation to audit the subsequent change to clinical practice.

### Messages for future care

In comparison to the local reviews, many additional, detailed messages for care were identified in the external reviews (table 3). Importantly, the external reviews highlighted aspects of good care in every case (n=33, 100%) compared with only 55% (n=18) of the local reviews which identified good care (p<0.0005). The local reviews included individual disciplinary actions, for example, four recommended individual supervisory action; a need for discipline of specific individuals was not identified in any external reviews (p=0.11). For seven of the cases, local review reports noted local factors affecting the situation, such as ‘staff were dealing with two emergencies simultaneously’ and ‘insufficient room for resuscitation.’ External reviewers, who were making their assessment solely on the basis of the medical records of individual cases, did not identify any local factors. External reviewers suggested an alternative clinical approach in 16 cases, whereas alternative clinical approaches were not mentioned in any local reviews.

**Table 3:** Number of cases where themes were noted in local (n=28) and external reviews (n=33)

Theme	Local reviews N=28	External reviews N=33
National guidelines or good practice points:		
Followed	13	28
Not followed	11	24
Senior review mentioned		
Consultant involvement noted	14	28
Lack of consultant involvement	7	12
Communication themes reported		
Good:		
with the patient and family	2	19
between staff members	4	14
Poor:		
with the patient and family	3	9
between staff members	4	11
Comments on documentation		
Some areas of good quality	8	13
Some areas of poor quality	10	29
Medication errors noted	2	11
Individual supervisory action recommended	4	0
Reported delays in:		
Diagnosis	5	22
Obtaining a second opinion	2	3
Providing treatment (excluding drugs)	7	9
Administering drugs (eg, antibiotics)	3	5
Requesting blood or clotting products	0	2
Gaining access to blood or clotting products	0	2
Transferring patients (eg, to theatre or the labour ward)	2	6
Suggestions for alternative clinical approach made	0	16

Note that several themes may have been identified for each woman, and therefore the total number of themes identified exceeds the actual number of cases examined.

While the external reviewers made a judgement that five (15%) women had received ‘good care,’ ‘improvements to care that would not have made a difference to outcome’ were identified in 17 cases (52%), and for eleven women (33%), ‘improvements to care were identified that may have made a difference to outcome.’ These improvements which might have changed the women's outcome had only been identified in the local reviews for four of these cases.

### Examples of lessons identified

Overall, the lessons for care identified in the local reviews were briefer and less focused than those identified on external reviews. The following are examples of lessons identified from the local review of the care of a woman with a postpartum haemorrhage and hysterectomy:

- Syntometrine not indicated.
- Incorrect dose of syntocinon.
- Incorrect practice.
- [Long] timescale between delivery of placenta and examination.
- No notes from Registrar.
- No ongoing estimation of blood loss.
- Guidelines followed once gynae[cology] consultant arrived.
- ?Incorrect estimation of blood loss.
- Timely decision for hysterectomy.
- Appropriate place for care.

In contrast, the external assessor’s reports into the same case gave more detailed comments:

- Care provided to this woman appears to have been extremely well managed (National Institute for Health and Care Excellence (NICE) Guidance: Routine care for the healthy pregnant woman. http://www.nice.org.uk/Guidance/CG62).
- The multidisciplinary team appears to have responded quickly to the emergency situation.
- Consultant obstetricians and anaesthetists were actively involved in the care.
- In addition to the measures taken to control the haemorrhage, other procedures should have been considered at or before laparotomy to preserve the uterus. The notes do not reflect consideration of tying off the uterine arteries, calling for vascular surgical assistance or considering uterine artery embolisation (Prevention and Management of postpartum haemorrhage. http://www.rcog.org.uk/files/rcog-corp/GT52PostpartumHaemorrhage0411.pdf). I am unaware of the facilities available in the unit, and if any of these measures could have been taken but potentially, this would have prevented the need for hysterectomy.
- The resuscitation was excellent, including ensuring active warming and temperature monitoring.
- The medical notes are adequate but do not give a very clear picture of the events and the decision process at each stage. Lack of observation charts.

The lessons identified in a case of maternal sepsis illustrate the different focus observed among local compared with external reviews. The local review of the case noted the following points with respect to suggestions for local service improvements:

- Delay in the administration of antibiotics within the ‘golden hour’ [the hour immediately after sepsis is first suspected].
- Consultant obstetrician informed who discussed the incident with consultant on [Accident and Emergency] (A&E).
- All pregnant women over 20 weeks gestation who attend A&E should be transferred to either the delivery suite or patient assessment unit (except those involved in major trauma when A&E should call the obs[tetrics] and gynae[cology] team to attend A&E).
- Feedback to A&E staff and include in communications diary.
- Raise awareness with [Ambulance Service] advising that all women of 20 weeks and over should be brought straight to maternity.

In contrast, the external reviews of the same case focused more on specific aspects of clinical care and noted wider actions in the whole care pathway (including the general practitioner (GP)):

- There were delays in recognition and management of this woman's sepsis.
- Only two of the six ‘sepsis six bundle’ measures were implemented by A&E staff—blood and oxygen therapy, and there was a significant delay in commencing IV antibiotics.
- The ‘Surviving Sepsis’ campaign highlights the importance of early antibiotic therapy in the ‘golden hour’ after presentation.
- Pregnancy should not be a reason to postpone antibiotic administration when sepsis is suspected, and neither is it a contraindication to chest X-ray.
- A [Modified Early Warning Score] (MEWS) of 7 should generate a more urgent medical assessment.
- There might have been some delay in recognising the severity of her illness by her GP, as it was documented that she had been ill for 4–6 weeks.

There were several cases in which local reviews identified no lessons for care but where external reviews considered that there were important lessons to be learned. For example, the following was noted from local assessment of the care of a woman with uterine inversion:

- All appropriate actions had been undertaken and no reason could be found for it to have occurred, and therefore, no formal report was completed.

In contrast, the external reviewers noted clear lessons from reviewing the same woman's care:

- The management of this woman's labour and delivery was complicated by the fact that she was not suitable for regional anaesthesia. There seems to be little planning for analgesia in labour or if anaesthesia were required at delivery (the option of central neuraxial blockade having been dismissed).
- The anaesthetist however did not recommend any alternative options, nor did an anaesthetist review this woman in labour when pain management was clearly an issue. This led to a delay in the first stage of labour because there was reluctance to commence syntocinon (despite this being a primiparous woman's induction) as this would strengthen contractions and consequently pain.
- There was also a delay in the second stage (over 4 h), as although earlier delivery was considered it was thought a better option to allow this woman to continue pushing as vaginal examination was so difficult due to her distress.
- The alternative at this time was a general anaesthetic for a forceps delivery in theatre.
- Uterine inversion occurred within 9 min of delivery of the baby; so, almost certainly mismanagement of third stage.
- I am sure delivery was challenging for all concerned. There was immediate recognition that uterine inversion had occurred, and it was dealt with and managed appropriately.
- Query whether the patient was debriefed or whether the significance for future pregnancies was discussed.

### Costs of external reviews

The administrative time spent on organising, photocopying, anonymising and scanning each case and liaising with external assessors was a mean of 17 h, which amounts to an average administrative cost of £300 per case. On average it took each external assessor 3 h to read and report on each case. The cost of five external assessors' time, based on the estimated consultant and senior midwife salary costs,^13^ was £1800 per case. Thus, the total cost of conducting an external review into a near-miss case of maternal morbidity per case was estimated as £2100.

### Feedback from external reviewers

All 11 external reviewers completed the questionnaire to provide feedback on the process. The common challenges faced with conducting external reviews were the poor quality of the notes, working in isolation and having time to do the reviews, as well as meeting the requirements of NHS work.

The reviewers viewed their roles as external assessors in this study and as local assessors in their own maternity units to be different. An external assessor’s role was thought to be to provide an unbiased, objective opinion on the clinical care documented. As local assessors, they would evaluate skills among staff, training, protocols and organisational issues. Some found it quicker and easier to be involved in local reviews than external reviews because of being able to speak to the staff involved and having access to all the notes. The reported challenges of being involved in local reviews were being influenced by the people involved in an incident and giving constructive criticism to colleagues while avoiding a blame culture.

### Feedback from local staff

Feedback was obtained in questionnaires from 75% (36 of 48) of the members of local staff from the six participating maternity units who attended the feedback sessions. The three key points to optimise local review that were most frequently recalled from the discussion were the importance of multidisciplinary review, the need to understand the local situation at the time of the incident, and ensuring lessons are learned and implemented in a constructive manner. Many members of staff reported appreciation of the educational benefits gained from reviewing incidents following presentation of the results of the study.

Some improvements to local reviews had already been put in place following the presentation of the results of the study. Staff from one unit reported that anaesthetists were now invited to their incident review meetings, and several individuals reported that they were more conscious of improving their documentation in clinical notes and in the minutes of local review meetings. However, almost half of the local staff members (n=16) reported that no changes had been made to the clinical practice in their unit following the presentation of the results.

## Discussion

The local reviews of the care of women with severe pregnancy complications were very varied. A formal report was written for only one-third, and very few used a recognised methodology such as root cause analysis. Only one of the reviews included in the study was multidisciplinary, involving midwives, obstetricians and anaesthetists. Patients were involved in fewer than one in six reviews. An action plan was drawn up for fewer than half the cases examined; one-fifth of the action plans included recommendations for auditing changes in practice, as a result of the lessons for care identified in the review. External reviews of the same cases identified many additional messages for care; the most notable differences were that external reviews identified examples of good care in every woman's case, and that there were no external reviews that recommended individual disciplinary procedures for staff. External reviewers identified alternative recommended management approaches in half the cases.

Recent reports of critical incidents in maternity units in the UK have identified similar themes of mixed purposes to reviews and lack of local critical review.^7^ External review (confidential enquiry) is carried out nationally using a standard approach.^4^ The observed differences between local and external reviews could be due to the uniform and standardised nature of the external reviews and the fact that the assessors received common online training prior to undertaking the assessments. Improvements in local reviews may thus be achieved by local assessors undertaking similar training or using national protocols. This would allow difficult areas such as cultural issues to be addressed, to allow unbiased, detailed positive and constructive feedback.

In this comparison of local and external reviews, we were unable to establish whether the external or the multiprofessional perspective added to the lessons learned and therefore the apparent advantages of external reviews. Both local and external review processes identified important messages to improve future care, although the number of specific messages identified was greater in the external reviews. Although there was little evidence of multidisciplinary review at a local level, it is important to note that it is possible that the review reports did not fully capture the multidisciplinary nature of discussions that might have occurred outside of the formal review meetings. It was also apparent that local review groups had a role to institute individual disciplinary procedures, where required, which may detract from the identification of generalised messages to improve clinical care.

However, the external review process is labour-intensive, requires administrative support and on average we estimate it would cost £2100 per case. We were not able to capture costs for the local review processes. Such additional costs are likely to prohibit the use of external reviews in current maternity practice and emphasise the importance of focusing on optimising local review processes. Nevertheless, while the costs appear high, if lessons are learned and implemented, future serious morbidity could be prevented. In addition to patient benefit, this would also potentially prevent future litigation costs. These costs were estimated by the National Audit Office in 2013 as £700 out of the £3700 spent on average on each birth in England.^14^

This study included only six units, randomly selected from all units in England, stratified by size and risk management (CNST) level, and thus they may not be representative of maternity units in England as a whole. Nevertheless, we identified many differences in the review processes used at a local level even among this limited number of units, and we have no reason to suspect that this pattern of variation in methods used, and professional groups involved, would not be replicated across all maternity units. No copies of electronic records (eg, antenatal, postnatal and intensive care unit notes) or local protocols were available to the external assessors, and thus the external reviews could only be based on the documentation provided by the maternity units. The cases assessed were mostly cases of postpartum haemorrhage, since this represents the most common severe complication in maternity care,^15^ and thus it is possible that the differences we observed between local and external reviews would not be the same if the care of women with other morbidities were examined. However, the classification of care received by the women in this study, who predominantly had postpartum haemorrhage, was similar to the classification of care for the women who survived septic shock who were included in the recent MBRRACE-UK confidential enquiry,^4^ which gives some evidence that the results may be generalisable to other conditions.

Substantial variability in local reviews is consistent with the findings of a national survey that documented considerable disparity in the types of incident listed for review by maternity units in the UK, including maternal sepsis only being listed by two-thirds of units, despite this being a current major concern within the NHS and internationally.^4^ ^16^ ^17^ A systematic appraisal of the quality of local guidelines for incident reviews in UK maternity units concluded that guidance was of good or high quality for 55%, and that 81% recommended that a multidisciplinary group of health professionals should review incidents.^18^ This guidance does not appear to be reflected in the actual practice identified in this study, since the majority of local reviews were not multidisciplinary. It was striking to note that only three local reports included mention of monitoring or audits of changes in clinical practice in response to review recommendations. Almost three quarters of local incident review guidelines recommend such auditing,^18^ which is essential to monitor the implementation and impact of recommendations.

Although this work focuses on incident reviews in maternity, following on from the high-profile failures documented in recent reports,^7^ other authors have highlighted problems with current local incident review processes throughout the healthcare system.^19^ ^20^ Particular differences in the safety review and reporting processes which have grown up within the healthcare system, in comparison to the aviation industry from which they were derived, such as a focus on quantity rather than quality of incident review reports,^19^ are echoed in the findings of our study. Our observation that local reviewers appear to have a role for instituting individual disciplinary procedures is also at odds with the principle from other industries that incident reporting is coordinated by an operationally independent group.^19^ ^20^ This, perhaps, argues for the more widespread use of the Confidential Enquiry process, as it is, by design, conducted by an independent group.^5^ Other criticisms of current healthcare incident reporting processes have focused on the fact that the focus is on reporting rather than in-depth investigation and driving improvement,^19^ a feature also reflected in the local reviews we examined, in which fewer than 1 in 10 planned to audit the recommended changes in practice.

## Conclusions

The clear message from this study is that the quality of local reviews of the care of women with severe maternal morbidity can be improved. Very few of the reviews involved patients, despite recent initiatives to improve transparency in examining the quality of care. Local reviews should be multidisciplinary, and should be managed as a separate process to those that include individual disciplinary procedures. Local reviews should generate an action plan, and the implementation of recommendations should be audited to ensure that change has led to the desired improvement in outcomes. Improvements in local reviews may be achieved by local assessors undertaking standardised training or the development of national review protocols. Further evaluation is needed to establish whether there is added value to including an external perspective to local reviews once high-quality multidisciplinary reviews are fully implemented.
